# Supplementary material for: An updated nuclear-physics and multi-messenger astrophysics framework for binary neutron star mergers
Source: Nat Commun. 2023 Dec 20;14:8352. doi: 10.1038/s41467-023-43932-6 (PMC10733434; doi:10.1038/s41467-023-43932-6)
Supplement: Supplementary file 1 — Supplementary Information [file 41467_2023_43932_MOESM1_ESM.pdf]

Supplementary information for "An updated nuclear-physics and multi-messenger astrophysics framework for binary neutron star mergers"

Peter T. H. Pang[1,2], Tim Dietrich[3,4], Michael W. Coughlin[5], Mattia Bulla[6,7,8,9], Ingo Tews[10], Mouza Almualla[11], Tyler Barna[5], Ramodgwendé Weizmann Kiendrebeogo[12,13], Nina Kunert[3], Gargi Mansingh[5,14], Brandon Reed[5,15], Niharika Sravan[16], Andrew Toivonen[5], Sarah Antier[13], Robert O. VandenBerg[5], Jack Heinzel[17], Vsevolod Nedora[4], Pouyan Salehi[3], Ritwik Sharma[18], Rahul Somasundaram[10,19,20], Chris Van Den Broeck[1,2]

[1]Nikhef, Science Park 105, 1098 XG Amsterdam, The Netherlands [2]Institute for Gravitational and Subatomic Physics (GRASP), Utrecht University, Princetonplein 1, 3584 CC Utrecht, The Netherlands [3]Institut für Physik und Astronomie, Universität Potsdam, Haus 28, Karl-Liebknecht-Str. 24/25, 14476, Potsdam, Germany [4]Max Planck Institute for Gravitational Physics (Albert Einstein Institute), Am Mühlenberg 1, Potsdam 14476, Germany [5]School of Physics and Astronomy, University of Minnesota, Minneapolis, Minnesota 55455, USA [6]The Oskar Klein Centre, Department of Astronomy, Stockholm University, AlbaNova, SE-106 91 Stockholm, Sweden [7]Department of Physics and Earth Science, University of Ferrara, via Saragat 1, I-44122 Ferrara, Italy [8]INFN, Sezione di Ferrara, via Saragat 1, I-44122 Ferrara, Italy [9]INAF, Osservatorio Astronomico d'Abruzzo, via Mentore Maggini snc, 64100 Teramo, Italy [10]Theoretical Division, Los Alamos National Laboratory, Los Alamos, NM 87545, USA [11]Department of Physics, American University of Sharjah, PO Box 26666, Sharjah, UAE [12]Laboratoire de Physique et de Chimie de l'Environnement, Université Joseph KI-ZERBO, Ouagadougou, Burkinka Faso [13]Observatoire de la Côte d'Azur, Université Côte d'Azur, CNRS, 96 boulevard del'observatoire, F06304 Nice cedex 4, France [14]Department of Physics, American University, Washington, DC 20016, USA. [15]Department of Physics and Astronomy, University of Minnesota – Duluth, Duluth, MN 55812 [16]Department of Physics, Drexel University, Philadelphia, PA 19104, USA [17]Department of Physics, Massachusetts Institute of Technology, 77 Massachusetts Ave, Cambridge, MA 02139, USA. [18]Department of Physics, Deshbandhu College, University of Delhi, New Delhi, India. [19]Univ Lyon, Univ Claude Bernard Lyon 1, CNRS/IN2P3, IP2I Lyon, UMR 5822, F-69622, Villeurbanne, France [20]Department of Physics, Syracuse University, Syracuse, NY 13244, USA

1 Technical Framework

Computing generic kilonova lightcurves.

Gaussian Process Regression: Because we are given vectors of photometry or spectra from radiative transfer simulations, we require methods to interpolate between these grids. If we denote the parameters of the models as $\mathbf{\Theta}^j$ and the vectors of data as τ, parameterized by the i-th time index, we can create matrices of simulations as $\mathcal{T}_{ij} = [\tau_i(\mathbf{\Theta}^j)]$. From these matrices, there are a variety of ways to interpolate these vectors, including direct interpolation of the vectors. Here, instead, we interpolate the principal components of each τ_i. To compute the principal components, we take the singular value decomposition (SVD) of this matrix

$$\mathcal{T} = V\Sigma U^\top. \tag{1}$$

The SVD computes orthonormal basis vectors in the columns and rows of V and U. With this new basis, we can project our original τ_i onto the left-singular vector basis

$$s_k(\boldsymbol{\theta}^j) = V_{ki}^\top \tau_i(\boldsymbol{\theta}^j), \tag{2}$$

where s_k are the weights of the principal components of the input data $\mathcal{T}_{ij}$. This method has the benefit of maximizing the variance in each subsequent basis vector, meaning we can truncate this sum to minimize computational resources; in our case, we find that using the first 10 basis vectors is sufficient for a reliable representation of the lightcurves.

To interpolate the principal component eigenvalues, we use Gaussian process regression (GPR) [1], which relies on the assumption that the correlation between neighboring values can be represented by a multivariate Gaussian distribution. The covariance between function values is known as a "kernel" function, with many kernel functions commonly used in the literature; in our analysis, we use a rational-quadratic kernel function implemented in `sci-kit learn` [2]:

$$k(\boldsymbol{\theta}^i, \boldsymbol{\theta}^j) = \left(1 + \frac{d(\boldsymbol{\theta}^i, \boldsymbol{\theta}^j)^2}{2\alpha l^2}\right)^{-\alpha} \tag{3}$$

where $\alpha = 0.1$ and $l = 1.0$ for our implementation.

As is common in GPR-based interpolation, we first normalize the components of $s_k(\boldsymbol{\theta}^j)$ by subtracting the minimum value and dividing by the difference between the maximum and minimum value. After the interpolation, the de-whitened values are projected back into the time domain:

$$\tau_i(\boldsymbol{\theta}^j) = V_{ik} s_k(\boldsymbol{\theta}^j) \tag{4}$$

Finally, the interpolated $\tau_i(\boldsymbol{\theta}^j)$ is then used for the computation of the likelihood.

Supplementary Table 1 | Posterior of and prior used for the GW170817-and-AT2017gfo and GW170817-and-AT2017gfo-GRB170817A analysis.

Parameter	Name	Connection with other parameters	prior	Posterior
m_i	Component mass	-	$\mathcal{U}(0.5M_\odot, 4.3M_\odot)$	$m_1 = 1.41^{+0.05}_{-0.05} M_\odot$<br>$m_2 = 1.31^{+0.04}_{-0.04} M_\odot$
EOS	Equation-of-state of nuclear matter	-	$\mathcal{U}(1, 5000)$	EOS = $4734.09^{+253.85}_{-304.07}$
$\mathbf{s}_i$	Component spin	-	Uniform on a sphere with $\lvert\mathbf{s}_i\rvert < 0.05$	$\mathbf{s}_1 = (0.00^{+0.02}_{-0.02}, 0.00^{+0.02}_{-0.02}, 0.00^{+0.01}_{-0.01})$<br>$\mathbf{s}_2 = (0.00^{+0.02}_{-0.02}, 0.00^{+0.02}_{-0.02}, 0.00^{+0.01}_{-0.01})$
Λ_i	Tidal deformability	$\Lambda(m_i; \mathrm{EOS})$	-<br>-	$\Lambda_1 = 352.71^{+107.84}_{-114.83}$<br>$\Lambda_2 = 530.26^{+135.03}_{-116.34}$
C_i	Compactness	$C_i = C(m_i; \mathrm{EOS})$	-<br>-	$C_1 = 0.17^{+0.01}_{-0.01}$<br>$C_2 = 0.16^{+0.01}_{-0.01}$
α	Dynamical ejecta mass fitting error	-	$\mathcal{N}(0M_\odot, 0.0004M_\odot)$	$\alpha = -0.00005^{+0.0005}_{-0.0006} M_\odot$
$m^{\mathrm{ej}}_{\mathrm{dyn}}$	Dynamical ejecta mass	$m^{\mathrm{ej}}_{\mathrm{dyn}} = m^{\mathrm{ej}}_{\mathrm{dyn,fit}}(m_i, C_i) + \alpha$	-	$\log_{10}(m^{\mathrm{ej}}_{\mathrm{dyn}}/M_\odot) = -2.25^{+0.10}_{-0.07}$
$M_{\mathrm{threshold}}$	Threshold mass	$M_{\mathrm{threshold}} = M_{\mathrm{threshold}}(\mathrm{EOS})$	-	$M_{\mathrm{threshold}} = 3.76^{+0.10}_{-0.09} M_\odot$
m_{disk}	Disk mass	$m_{\mathrm{disk}} = m_{\mathrm{disk}}(m_i, M_{\mathrm{threshold}})$	-	$\log_{10}(m_{\mathrm{disk}}/M_\odot) = -0.76^{+0.02}_{-0.01}$
ξ	Fraction of the disk mass ejected as wind	-	$\mathcal{U}(0, 1)$	$\xi = 0.61^{+0.18}_{-0.17}$
$m^{\mathrm{ej}}_{\mathrm{wind}}$	Wind ejecta mass	$m^{\mathrm{ej}}_{\mathrm{wind}} = \xi \times m_{\mathrm{disk}}$	-	$\log_{10}(m^{\mathrm{ej}}_{\mathrm{wind}}/M_\odot) = -1.18^{+0.08}_{-0.09}$
Φ	Lanthanide-rich composition opening angle	-	$U(15\mathrm{deg}, 75\mathrm{deg})$	$\Phi = 68.69^{+4.77}_{-4.61}\mathrm{deg}$
ϵ	Fraction of leftover disk mass converted to GRB jet energy	-	$\log\mathcal{U}(-7, -0.3)$	$\epsilon = 0.04^{+0.23}_{-0.04}$
E_0	GRB jet on-axis isotropic energy	$E_0 = \epsilon \times (1 - \xi) \times m_{\mathrm{disk}}$	$\log\mathcal{U}(48, 60)$	$\log_{10}(E_0/\mathrm{erg}) = 51.45^{+1.05}_{-1.24}$
θ_c	Half-width of the jet core	-	$\mathcal{U}(0.01\mathrm{rad}, \pi/2\mathrm{rad})$	$\theta_c = 0.37^{+0.60}_{-0.29}$ rad
θ_w	Truncation angle of the jet	-	$\mathcal{U}(0.01\mathrm{rad}, \pi/2\mathrm{rad})$	$\theta_w = 0.24^{+0.11}_{-0.09}$ rad
n_0	Number density of ISM	-	$\log\mathcal{U}(-6, 0)$	$\log_{10}(n_0/\mathrm{cm}^{-3}) = -3.96^{+1.62}_{-1.73}$
p	Electron distribution power-law index	-	$\mathcal{U}(2, 5)$	$p = 2.11^{+0.07}_{-0.06}$
ϵ_e	Thermal energy fraction in electrons	-	$\log\mathcal{U}(-4, 0)$	$\log_{10}\epsilon_e = -0.77^{+0.77}_{-1.05}$
ϵ_B	Thermal energy fraction in magnetic field	-	$\log\mathcal{U}(-5, 0)$	$\log_{10}\epsilon_B = -2.39^{+2.26}_{-1.64}$
$R_{1.4}$	Radius of a $1.4M_\odot$ neutron star	$R_{1.4}(\mathrm{EOS})$	-	$R_{1.4} = 11.98^{+0.35}_{-0.40}\mathrm{km}$

The tabke summarizes the intrinsic parameters for the multi-messenger observation of a BNS merger. In the fourth column, we report median posterior values at 90% credibility for the joint inference of GW170817-and-AT2017gfo-and-GRB170817A. $\mathcal{U}(a, b)$ refers to uniform distribution between a and b. $\log\mathcal{U}(a, b)$ refers to the log-uniform (of base 10) distribution, i.e. if $X \sim \log\mathcal{U}(a, b)$, $\log_{10} X \sim \mathcal{U}(a, b)$. $\mathcal{N}(\mu, \sigma)$ refers to a normal distribution with mean μ and variance of σ^2.

Neural Networks: Another alternative method for the grid interpolation is a feed-forward neural network (NN) which can predict the kilonova lightcurves based on the input parameters used by the chosen model [3]. The main advantage of this approach is it to reduce the memory footprint for the lightcurves computation.

To train the NN, we use about 2000 lightcurves computed with the full radiative transfer code POSSIS [4]. These lightcurves are split into a training data set of 90% and an evaluation dataset that contains 10% of all lightcurves. In the pre-processing stage, all the data was normalized between 0 and 1 via the usual MinMax normalization method, and similar to the GPR method, we used PCA data reduction to reduce the dimensions of the output parameter space to 10 components. In addition, we use simple linear interpolation for the cases in which the requested time interval is larger than the original in-hand data.

To develop our NN, we used Keras API from TENSORFLOW[5]. Our NN comprises an input layer with the same number of neurons as the input parameter space, three dense hidden layers with 64, 128, and 128 neurons each, and an output layer with ten neurons for the bolometric luminosity and the nine observational bands. We use the Adam optimizer with a learning rate of 0.01 and a Rectified linear unit as activation function. Finally, the NN is trained using a batch size of 32, epoch count of 15, and mean squared error (MSE) as loss function. To reconstruct the real lightcurves, a series of inverse transformations is applied with respect to the PCA and the normalization. Overall a MSE of 0.0022 is achieved.

EOS Sampling. For a set of EOSs, such as our sets constrained by chiral EFT, our framework is able to directly sample over EOSs instead of sampling over the masses and tidal deformabilities independently. Since these EOSs relate masses and tidal deformabilities based on nuclear-physics information, the tidal deformabilities can be computed for a given mass and EOS according to

$$p(\Lambda_i | m_i, \mathrm{EOS}) = \delta(\Lambda_i - \Lambda(m_i; \mathrm{EOS})). \tag{5}$$

This feature enables the possibility to include more physical information on the NS sources during parameter estimation ab initio and can be used through the installation of additional BILBY and PARALLEL BILBY patches that come along with our NMMA framework. Another advantage of this functionality is that information from multiple simulations can be combined to compute a combined posterior because the EOS is a common parameter for all NSs[7].

Combined Sampling. To extract most information from the GW and the EM data, we perform a full parameter estimation combining both likelihoods. The 'full' likelihood $\mathcal{L}$ is given by

$$\mathcal{L}(\boldsymbol{\theta}) = \mathcal{L}_{\mathrm{GW}}(\boldsymbol{\theta}_{\mathrm{GW}}) \times \mathcal{L}_{\mathrm{EM}}(\boldsymbol{\theta}_{\mathrm{EM}}), \tag{6}$$

where $\boldsymbol{\theta} = \{\boldsymbol{\theta}_{\mathrm{GW}}, \boldsymbol{\theta}_{\mathrm{EM}}\}$. Because the lightcurve models and the GW waveform models depend on different sets of parameters, simply sampling over all of the parameters would not yield a stronger constraint on the parameters of interest. Therefore, it is key to use connections between different parameters at the prior level. In particular, a few parameters describing the EM signals can be determined by the binary

Supplementary Table 2 | Posterior of and prior used for the GW170817-and-AT2017gfo and GW170817-and-AT2017gfo-GRB170817A analysis with the model from Ref.[6].

Parameter	Name	Connection with other parameters	prior	Posterior
m_i	Component mass	-	$\mathcal{U}(0.5M_\odot, 4.3M_\odot)$	$m_1 = 1.46^{+0.07}_{-0.08}M_\odot$
				$m_2 = 1.28^{+0.07}_{-0.06}M_\odot$
EOS	Equation-of-state of nuclear matter	-	$\mathcal{U}(1, 5000)$	EOS = $4686.33^{+289.78}_{-432.21}$
$\mathbf{s}_i$	Component spin	-	Uniform on a sphere	$\mathbf{s}_1 = (0.00^{+0.02}_{-0.02}, 0.00^{+0.02}_{-0.02}, 0.00^{+0.01}_{-0.01})$
			with $\lvert\mathbf{s}_i\rvert < 0.05$	$\mathbf{s}_2 = (0.00^{+0.02}_{-0.02}, 0.00^{+0.02}_{-0.02}, 0.00^{+0.01}_{-0.01})$
Λ_i	Tidal deformability	$\Lambda(m_i; \mathrm{EOS})$	-	$\Lambda_1 = 272.25^{+118.90}_{-117.36}$
			-	$\Lambda_2 = 594.23^{+178.28}_{-156.80}$
C_i	Compactness	$C_i = C(m_i; \mathrm{EOS})$	-	$C_1 = 0.18^{+0.01}_{-0.01}$
			-	$C_2 = 0.16^{+0.01}_{-0.01}$
α	Dynamical ejecta mass fitting error	-	$\mathcal{N}(0M_\odot, 0.0004M_\odot)$	$\alpha = -0.00002^{+0.0005}_{-0.0006}M_\odot$
$m^{\mathrm{ej}}_{\mathrm{dyn}}$	Dynamical ejecta mass	$m^{\mathrm{ej}}_{\mathrm{dyn}} = m^{\mathrm{ej}}_{\mathrm{dyn,fit}}(m_i, C_i) + \alpha$	-	$\log_{10}(m^{\mathrm{ej}}_{\mathrm{dyn}}/M_\odot) = -2.17^{+0.16}_{-0.13}$
$M_{\mathrm{threshold}}$	Threshold mass	$M_{\mathrm{threshold}} = M_{\mathrm{threshold}}(\mathrm{EOS})$	-	$M_{\mathrm{threshold}} = 3.05^{+0.08}_{-0.09}M_\odot$
m_{disk}	Disk mass	$m_{\mathrm{disk}} = m_{\mathrm{disk}}(m_i, M_{\mathrm{threshold}})$	-	$\log_{10}(m_{\mathrm{disk}}/M_\odot) = -0.93^{+0.18}_{-0.19}$
ξ	Fraction of the disk mass ejected as wind	-	$\mathcal{U}(0, 1)$	$\xi = 0.26^{+0.13}_{-0.12}$
$m^{\mathrm{ej}}_{\mathrm{wind}}$	Wind ejecta mass	$m^{\mathrm{ej}}_{\mathrm{wind}} = \xi \times m_{\mathrm{disk}}$	-	$\log_{10}(m^{\mathrm{ej}}_{\mathrm{wind}}/M_\odot) = -1.52^{+0.11}_{-0.11}$
$m^{\mathrm{ej}}_{\mathrm{tot}}$	Total ejecta mass	$m^{\mathrm{ej}}_{\mathrm{tot}} = m^{\mathrm{ej}}_{\mathrm{dyn}} + m^{\mathrm{ej}}_{\mathrm{wind}}$	-	$\log_{10}(m^{\mathrm{ej}}_{\mathrm{tot}}/M_\odot) = -1.43^{+0.09}_{-0.09}$
v^{ej}	Ejecta velocity	-	$\log\mathcal{U}(-1, -0.6)$	$\log_{10}(v^{\mathrm{ej}}/c) = -0.74^{+0.06}_{-0.07}$
X_{lan}	Lanthanide mass fraction	-	$\log\mathcal{U}(-5, -2)$	$\log_{10} X_{\mathrm{lan}} = -3.38^{+0.13}_{-0.12}$
ϵ	Fraction of leftover disk mass converted to GRB jet energy	-	$\log\mathcal{U}(-7, -0.3)$	$\epsilon = 0.01^{+0.12}_{-0.01}$
E_0	GRB jet on-axis isotropic energy	$E_0 = \epsilon \times (1 - \xi) \times m_{\mathrm{disk}}$	$\log\mathcal{U}(48, 60)$	$\log_{10}(E_0/\mathrm{erg}) = 51.33^{+1.17}_{-0.99}$
θ_c	Half-width of the jet core	-	$\mathcal{U}(0.01\mathrm{rad}, \pi/2\mathrm{rad})$	$\theta_c = 0.12^{+0.36}_{-0.06}$ rad
θ_w	Truncation angle of the jet	-	$\mathcal{U}(0.01\mathrm{rad}, \pi/2\mathrm{rad})$	$\theta_w = 0.42^{+0.28}_{-0.19}$ rad
n_0	Number density of ISM	-	$\log\mathcal{U}(-6, 0)$	$\log_{10}(n_0/\mathrm{cm}^{-3}) = -2.87^{+1.56}_{-1.53}$
p	Electron distribution power-law index	-	$\mathcal{U}(2, 5)$	$p = 2.11^{+0.06}_{-0.06}$
ϵ_e	Thermal energy fraction in electrons	-	$\log\mathcal{U}(-4, 0)$	$\log_{10}\epsilon_e = -0.61^{+0.61}_{-1.09}$
ϵ_B	Thermal energy fraction in magnetic field	-	$\log\mathcal{U}(-5, 0)$	$\log_{10}\epsilon_B = -2.44^{+1.85}_{-1.9}$
$R_{1.4}$	Radius of a $1.4M_\odot$ neutron star	$R_{1.4}(\mathrm{EOS})$	-	$R_{1.4} = 11.86^{+0.37}_{-0.49}\mathrm{km}$

The table summarizes the intrinsic parameters for the multi-messenger observation of a BNS merger using the kilonova model described in Ref. [6]. In the fourth column, we report median posterior values at 90% credibility for the joint inference of GW170817-and-AT2017gfo-and-GRB170817A. $\mathcal{U}(a, b)$ refers to uniform distribution between a and b. $\log\mathcal{U}(a, b)$ refers to the log-uniform (of base 10) distribution, i.e., if $X \sim \log\mathcal{U}(a, b)$, $\log_{10} X \sim \mathcal{U}(a, b)$. $\mathcal{N}(\mu, \sigma)$ refers to a normal distribution with mean μ and variance of σ^2.

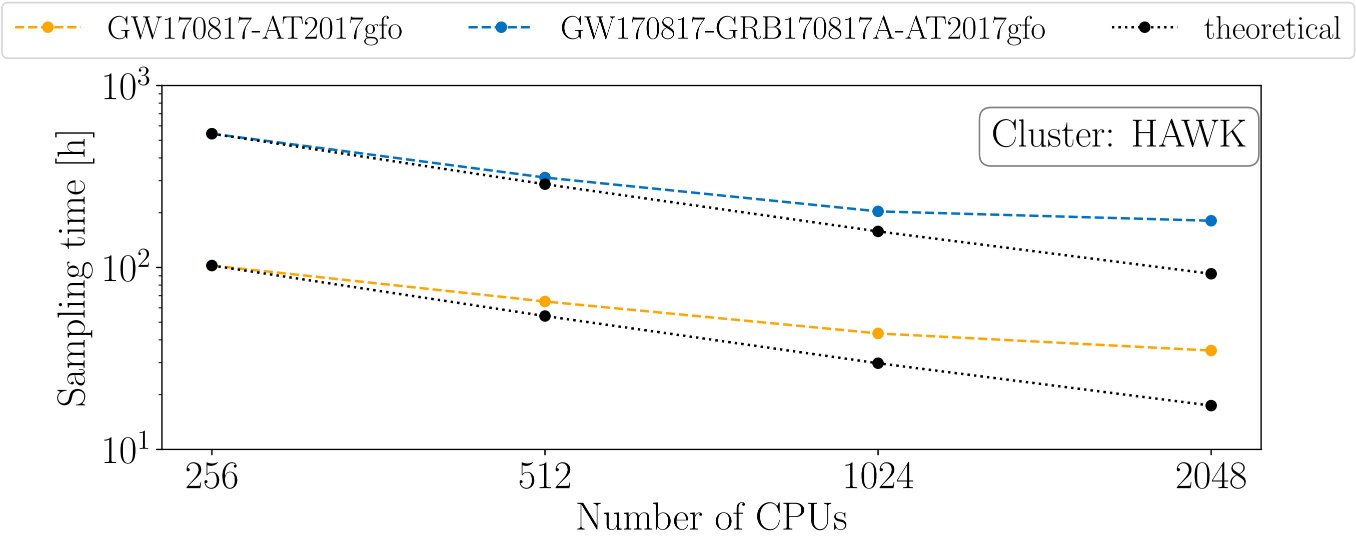

Supplementary Figure 1 | Scaling performance of the NMMA framework. Theoretical against measured scaling performance for the inference runs of GW170817-AT2017gfo and GW170817-GRB170817A-AT2017gfo on 256, 512, 1024, and 2048 cores, respectively. The theoretical scaling is computed for 256 cores using the reference time $T(N_{\mathrm{core}_{\mathrm{ref}}})$ = 102.49 h for GW170817-AT2017gfo and $T(N_{\mathrm{core}_{\mathrm{ref}}})$ = 543.45 h for GW170817-GRB170817A-AT2017gfo.

masses and the EOS. For instance, the dynamical ejecta mass $m^{\mathrm{ej}}_{\mathrm{dyn}}$ and the disk mass m_{disk} are connected to the binary properties through the relations discussed in the methods section. The details for all the parameters are summarized in Supplementary Tab. 1.

Scaling. Due to the enormous computational burden of simultaneously analysing both the EM and GW signals, we have to ensure a good parallelization of our code. For the NMMA framework, the parallelization is achieved by taking advantage of the flexibility of `dynesty` [8] and based on the interface presented in `parallel_bilby` [9].

In particular, the nested sampling process is parallelized using a head/worker strategy. The "head" organizes the live/dead points, and validates if the stopping criteria are reached, while the "workers" find new live points under the likelihood constraint. The theoretical scaling performance of such a strategy is given by [10]

$$T(N_{\mathrm{cores}}) = T(N_{\mathrm{core}_{\mathrm{ref}}}) \times \frac{\ln(1 + N_{\mathrm{core}_{\mathrm{ref}}}/n_{\mathrm{live}})}{\ln(1 + N_{\mathrm{cores}}/n_{\mathrm{live}})}, \tag{7}$$

where $T(N_{\mathrm{cores}})$ is the runtime using N_{cores} cores and $T(N_{\mathrm{core}_{\mathrm{ref}}})$ is the reference time using $N_{\mathrm{core}_{\mathrm{ref}}}$ cores. The parameter n_{live} denotes the number of live points.

To validate if such a scaling is achieved, we performed intensive scaling tests on SuperMUC_NG at the Leibniz Supercomputing Centre (Munich), Lise and Emmy of the North German Supercomputing Alliance, and on HAWK of the High-Performance Computing Center Stuttgart. We show results for scaling tests performed on HAWK using AMD EPYC 7742 processors. The tests are based on a full joint inference of GW170817-and-AT2017gfo and GW170817-and-GRB170817A-and-AT2017gfo which includes intermediate checkpointing and all necessary I/O-operations. The strong scaling for such simulations is shown in Supplementary Fig. 1 and is compared to the theoretical scaling mentioned of Eq. (7).

Modelling Uncertainty. Overall, the obtained multi-messenger constraints depend noticeably on the robustness and accuracy of the individual GW, kilonova, and GRB afterglow models but also on the uncertainty and accuracy of the underlying equations of state and nuclear physics computations. In the past, there have been numerous studies considering the effect of GW-model uncertainties, e.g., [7,11,12] and we have already employed multiple GW models in one of our previous studies also to investigate uncertainties with the conclusion that with the current amount of observational data, we are mainly limited by statistical and not systematic uncertainties [13]. As an example of the influence of the particular choice of the kilonova model, we employ two different kilonova models with different assumptions about the geometry and composition of the ejecta. In particular, we compare the results for our standard kilonova model as presented in Supplementary Tab. 1 with the results based on the model of Kasen et al. [6] in Supplementary Tab. 2. While we find that individual parameters can be different, we find a similar neutron star radius of $R_{1.4} = 11.86^{+0.37}_{-0.49}$km.

Effect of systematic uncertainty budget To investigate the effect of the value chosen for the systematics uncertainty budget, the analysis on the GW170817-AT2017gfo-GRB170817A event has been conducted with three different values of σ_{sys}, namely, 0.5mag, 1mag and 2mag.

To gauge the effect on the final equation-of-state constraint, the resulting posterior of the radius of a $1.4M_{\odot}$ neutron star, $R_{1.4}$, for these different σ_{sys} are compared. The median values with the 90% credible interval as uncertainties, are shown in Supplementary Tab. 3.

With lower values of σ_{sys}, the estimated $R_{1.4}$ is skewed towards higher values, with the minimal uncertainty at 1mag. Therefore, it hints that

- the information from difference channels are more coherent at 1mag as compare to 0.5mag
- the information is not over diluted in 1mag as compare to 2mag.

Supplementary Table 3 | Comparison of radius measurements of a $1.4M_\odot$ neutron star for different $\sigma_{\rm sys}$.

$\sigma_{\rm sys}$ [mag]	$R_{1.4}$ [km]
0.5	$12.05^{+0.35}_{-0.45}$
1.0	$11.98^{+0.35}_{-0.40}$
2.0	$11.76^{+0.41}_{-0.49}$

The resulting radius measurements of a $1.4M_\odot$ neutron star for $\sigma_{\rm sys}$ being 0.5, 1.0 and 2.0 are shown.

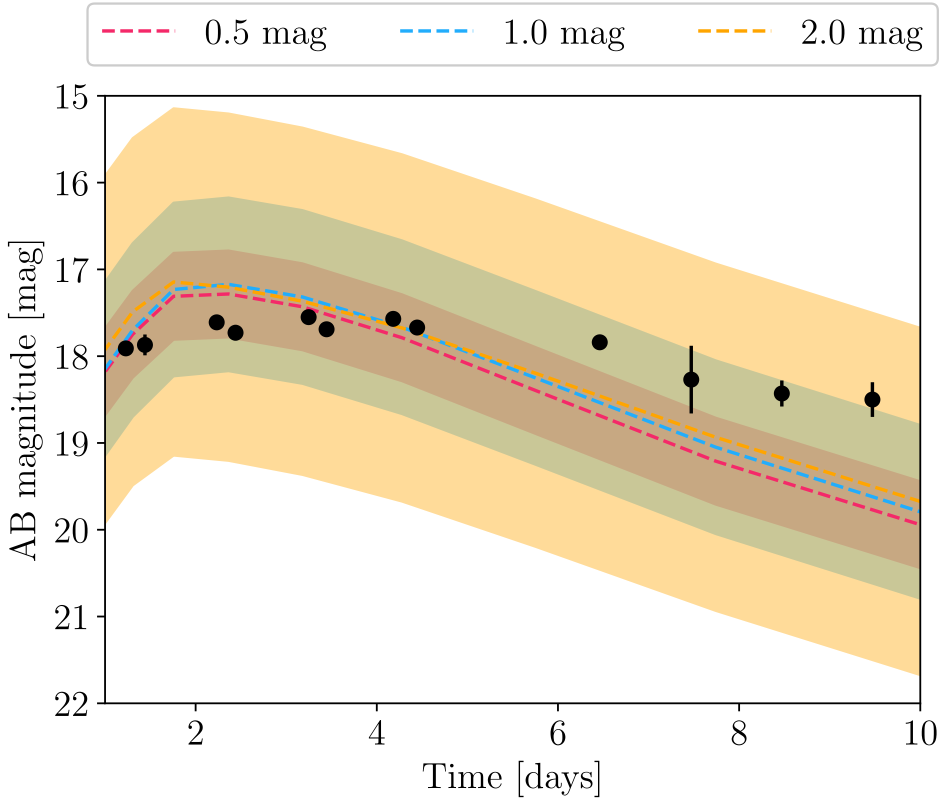

Supplementary Figure 2 | **Best-fit early-time lightcurve from the analysis with various $\sigma_{\rm sys}$.** The best-fit K-filter lightcurve (dashed, with the one magnitude uncertainty shown as the band) for AT2017gfo data when analysing GW170817-and-AT2017gfo-and-GRB170817A simultaneously with different $\sigma_{\rm sys}$.

To verify the above claim, we investigate into the estimated best-fit lightcurves. In particular, the best-fit lightcurves with the associated error budget for filter K is shown at Supplementary Fig. 2. One can see that the 0.5mag is underestimating the systematic uncertainty and failed to account for the data after 6 days after the merger, while the 2mag is overcompensating for the systematic uncertainty.

Based on the above observations, we concluded that the $\sigma_{\rm sys}$ of 1mag is a sensible choice.

1. Rasmussen, C. E. & Williams, C. K. I. *Gaussian Processes for Machine Learning* (MIT Press, 2006).
2. Pedregosa, F. *et al.* Scikit-learn: Machine learning in Python. *Journal of Machine Learning Research* **12**, 2825–2830 (2011).
3. Almualla, M. *et al.* Using Neural Networks to Perform Rapid High-Dimensional Kilonova Parameter Inference. *arXiv:2112.15470* (2021).
4. Bulla, M. POSSIS: predicting spectra, light curves and polarization for multi-dimensional models of supernovae and kilonovae. *Mon. Not. Roy. Astron. Soc.* **489**, 5037–5045 (2019). 1906.04205.
5. Abadi, M. *et al.* TensorFlow: Large-scale machine learning on heterogeneous systems (2015). URL https://www.tensorflow.org/. Software available from tensorflow.org.
6. Kasen, D., Metzger, B., Barnes, J., Quataert, E. & Ramirez-Ruiz, E. Origin of the heavy elements in binary neutron-star mergers from a gravitational wave event. *Nature, 10.1038/nature24453* (2017). 1710.05463.
7. Kunert, N., Pang, P. T. H., Tews, I., Coughlin, M. W. & Dietrich, T. Quantifying modeling uncertainties when combining multiple gravitational-wave detections from binary neutron star sources. *Phys. Rev. D* **105**, L061301 (2022). 2110.11835.
8. Speagle, J. S. dynesty: a dynamic nested sampling package for estimating Bayesian posteriors and evidences. *Mon. Not. Roy. Astron. Soc.* **493**, 3132–3158 (2020). 1904.02180.
9. Smith, R. J. E., Ashton, G., Vajpeyi, A. & Talbot, C. Massively parallel Bayesian inference for transient gravitational-wave astronomy. *Mon. Not. Roy. Astron. Soc.* **498**, 4492–4502 (2020). 1909.11873.
10. Handley, W., Hobson, M. & Lasenby, A. PolyChord: nested sampling for cosmology. *Mon. Not. Roy. Astron. Soc.* **450**, L61–L65 (2015). 1502.01856.
11. Samajdar, A. & Dietrich, T. Waveform systematics for binary neutron star gravitational wave signals: effects of the point-particle baseline and tidal descriptions. *Phys. Rev.* **D98**, 124030 (2018). 1810.03936.
12. Samajdar, A. & Dietrich, T. Waveform systematics for binary neutron star gravitational wave signals: Effects of spin, precession, and the observation of electromagnetic counterparts. *Phys. Rev.* **D100**, 024046 (2019). 1905.03118.

13. Dietrich, T. *et al.* Multimessenger constraints on the neutron-star equation of state and the Hubble constant. *Science* **370**, 1450–1453 (2020). 2002.11355.
